# 5-methoxytryptophan protects MSCs from stress induced premature senescence by upregulating FoxO3a and mTOR

**DOI:** 10.1038/s41598-017-11077-4

**Published:** 2017-09-11

**Authors:** Tzu-Ching Chang, Min-Fen Hsu, Chiu-Yueh Shih, Kenneth K. Wu

**Affiliations:** 1 Metabolomic Medicine Research Center China Medical University Hospital, Taichung, Taiwan; 2 Graduate Institute of Biomedical Sciences, China Medical University, Taichung, Taiwan; 3 Institute of Cellular and System Medicine, National Health Research Institutes, Zhunan, Taiwan

## Abstract

5-methoxytryptophan (5-MTP) is a newly discovered tryptophan metabolite which controls stress-induced inflammatory signals. To determine whether 5-MTP protects against stress-induced mesenchymal stem cell (MSC) senescence, we incubated bone marrow-derived MSC (BM-MSC) in high-glucose medium or regular medium for 2 weeks followed by addiction of 5-MTP (10 μM) or vehicle for 48 h. 5-MTP reduced p16 and p21 expression, senescence-associated β-Gal (SA-β-Gal) and IL-6 secretion and increased BrdU incorporation. 5-MTP exerted a similar effect on BM-MSC senescence induced by a sublethal concentration of H_2_O_2_. 5-MTP enhanced FoxO3a expression and increased superoxide dismutase and catalase activities in HG BM-MSCs. Silencing of FoxO3a with siRNA abrogated 5-MTP-mediated reduction of SA-β-Gal and IL-6 secretion but not p21 or p16. Since mechanistic target of rapamycin (mTOR) is involved in cellular senescence, we determined whether 5-MTP influences mTOR expression. Our data reveal that mTOR protein level was depressed in HG-MSC which was rescued by 5-MTP. Rapamycin abrogated 5-MTP-mediated suppression of p16, p21, SA-β-Gal and IL-6 and rise of BrdU incorporation. Our findings suggest that 5-MTP protects MSCs against stress-induced senescence via FoxO3a and mTOR upregulation and has potential to improve cell expansion for cell therapy.

## Introduction

Mesenchymal stromal cells (commonly known as mesenchymal stem cells, MSCs) are derived from adult human tissues such as bone marrow (BM) and adipose tissues or fetal tissues such as placenta, umbilical cord and cord blood. They are conventionally cultured in medium containing relatively low glucose concentration (1 gram/L, 5.5 mM). We previously reported that BM-MSCs cultured in a higher glucose (HG) concentration, i.e 25 mM for 2–4 weeks undergo senescence as manifested by large and flat cell morphology, growth arrest, and increased p16, p21, SA-β-Gal and interleukin-6 (IL-6)^1^. The senescence phenotypes are attenuated by pretreating BM-MSCs with anti-oxidants such as N-acetylcysteine suggesting that HG metabolic stress induces MSC senescence through generation of reactive oxygen species (ROS)^1^. ROS are considered to be a major culprit of cell senescence, aging and aging-related human disease^2–4^.

MSC senescence hampers tissue regeneration and repair through arrest of cell proliferation, secretion of pro-inflammatory cytokines and lysosomal abnormalities. Furthermore, premature cell senescence in culture restricts MSC expansion and limits its use in cell therapy. There is an urgent need to develop new strategies to control MSC senescence. In this study, we determined whether 5- methoxytryptophan (5-MTP) is effective in controlling MSC premature senescence. We recently reported the discovery of 5-MTP as a novel metabolite of L-tryptophan^5^. 5-MTP is produced in and released from several types of human cells including fibroblasts and vascular endothelial cells^5, 6^. 5-MTP is constitutively released into the extracellular milieu including the circulating blood^6^. Human serum contains detectable 5-MTP with a mean concentration of approximately 1 μM^6^. 5-MTP inhibits pro-inflammatory mediator-induced cyclooxygenase-2 (COX-2) expression and cytokine/chemokine release in macrophages^6^, blocks pro-inflammatory cytokine-induced adhesion molecules on endothelial cells^7^ and protects endothelial barrier function and vascular integrity^7^. We suspected that 5-MTP controls MSC senescence and senescence-associated secretory phenotype. To assess this possible action, we evaluated the effect of chemosynthetic pure L-5-MTP on HG metabolic stress- and oxidant stress-induced BM-MSC senescence. Our findings indicate that 5-MTP at μM concentrations attenuates HG- and H_2_O_2_-induced senescence phenotypes by upregulating FOXO3a and mTOR expressions.

## Results

### L-5-MTP prevents HG-induced senescence

**Figure 1:**
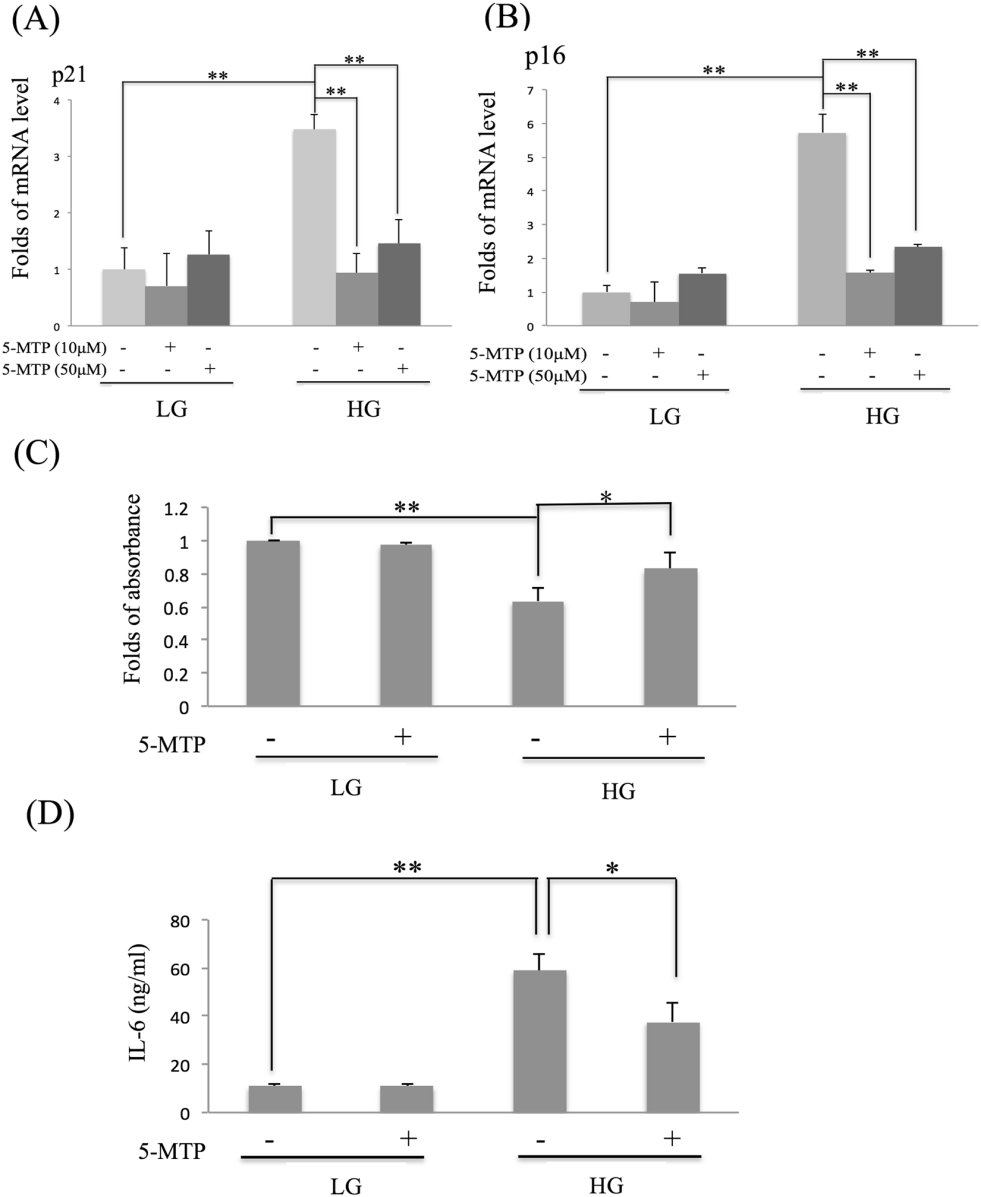
5-MTP protects BM-MSC from high glucose (HG) stress-induced cellular senescence. BM-MSCs were incubated in medium containing LG (5.5 mM) or HG (25 mM) for 2 weeks, chemosynthetic pure L-5-MTP (10 & 50 μM in A & B and 10 μM in C & D) was added into medium for 48 hrs before BM-MSCs were harvested and senescence markers were analyzed. **(A)** p21 and **(B)** p16 mRNA were analyzed by qPCR. **(C)** BrdU incorporation was analyzed as described in methods. **(D)** IL-6 released into the medium was measured by ELISA. Error bars in all figures denote means ± SEM (n = 3). *p < 0.05. **p < 0.01.

**Figure 2:**
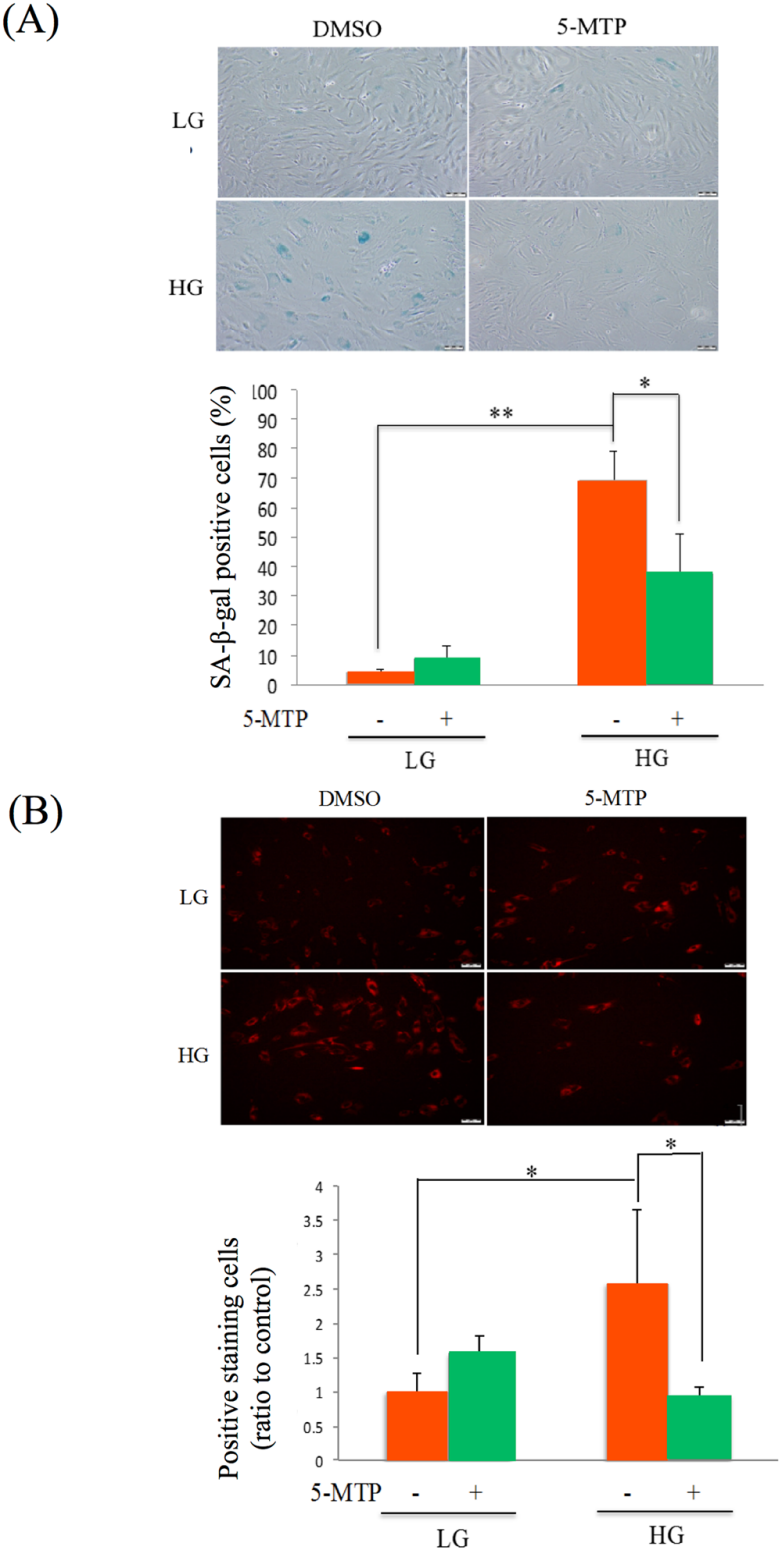
5-MTP suppresses HG stress-induced SA-β-gal and lysosome expansion in BM-MSCs. (**A)** After BM-MSCs had been cultured in LG or HG for 2 weeks they were incubated with or without 5-MTP (10 μM) for 48 hrs, washed and stained for SA-β-gal activity. SA-β-gal positive cells were quantified under light microscopy. Upper panel shows representative cell staining while lower panel quantitative analysis of SA-β-Gal positive cells. Error bars denote mean ± SEM (n = 3). *p < 0.05. **p < 0.01. **(B)** Lysosomes were detected by Lysotracker stain. Upper panel shows representative staining and the lower panel quantitative analyzes of cells with positive staining. Each bar denotes mean ± SEM of three experiments. *p < 0.05. **p < 0.01.

To explore the possible influence of 5-MTP on MSC senescence, we pretreated BM-MSCs cultured in the conventional medium containing low glucose (LG) concentration vs BM-MSCs cultured in HG with chemosynthetic pure L-5-MTP and analyzed changes in senescence phenotypes. A hallmark of cell senescence is growth arrest accompanied by an increased expression of cell cycle inhibitors, p16^INK4A^ and p21^WAF-1^. We analyzed p21 and p16 mRNA levels by qPCR. Positive and negative controls for p21 and p16 mRNA analysis are shown in Supplementary Fig. S1. 5-MTP prevented HG-induced p21 and p16 expression but did not affect p21 or p16 expression in LG-MSCs (Fig. 1A and B). BM-MSCs cultured in HG had a lower BrdU incorporation and pretreatment with 5-MTP alleviated the reduction (Fig. 1C). HG-induced IL-6 secretion was significantly reduced by 5-MTP pretreatment (Fig. 1D). 5-MTP did not exert a significant effect on SA-β-Gal in LG-MSCs while it significantly reduced HG-induced elevation of SA-β-Gal positive cells (Fig. 2A and Supplementary Fig. S2). As elevation of SA-β-Gal may be related to increased lysosome mass^8^, we analyzed lysosomes in HG- vs LG-BM-MSCs using Lysotracker Red staining. Lysotracker-positive cells were increased by HG which was reduced to the basal level by 5-MTP pretreatment (Fig. 2B and Supplementary Fig. S3). These results suggest that 5-MTP is active in blocking HG-induced senescence phenotypic changes and lysosome biogenesis.

### 5-MTP restores osteogenic and inhibits adipogenic differentiation induced by HG

**Figure 3:**
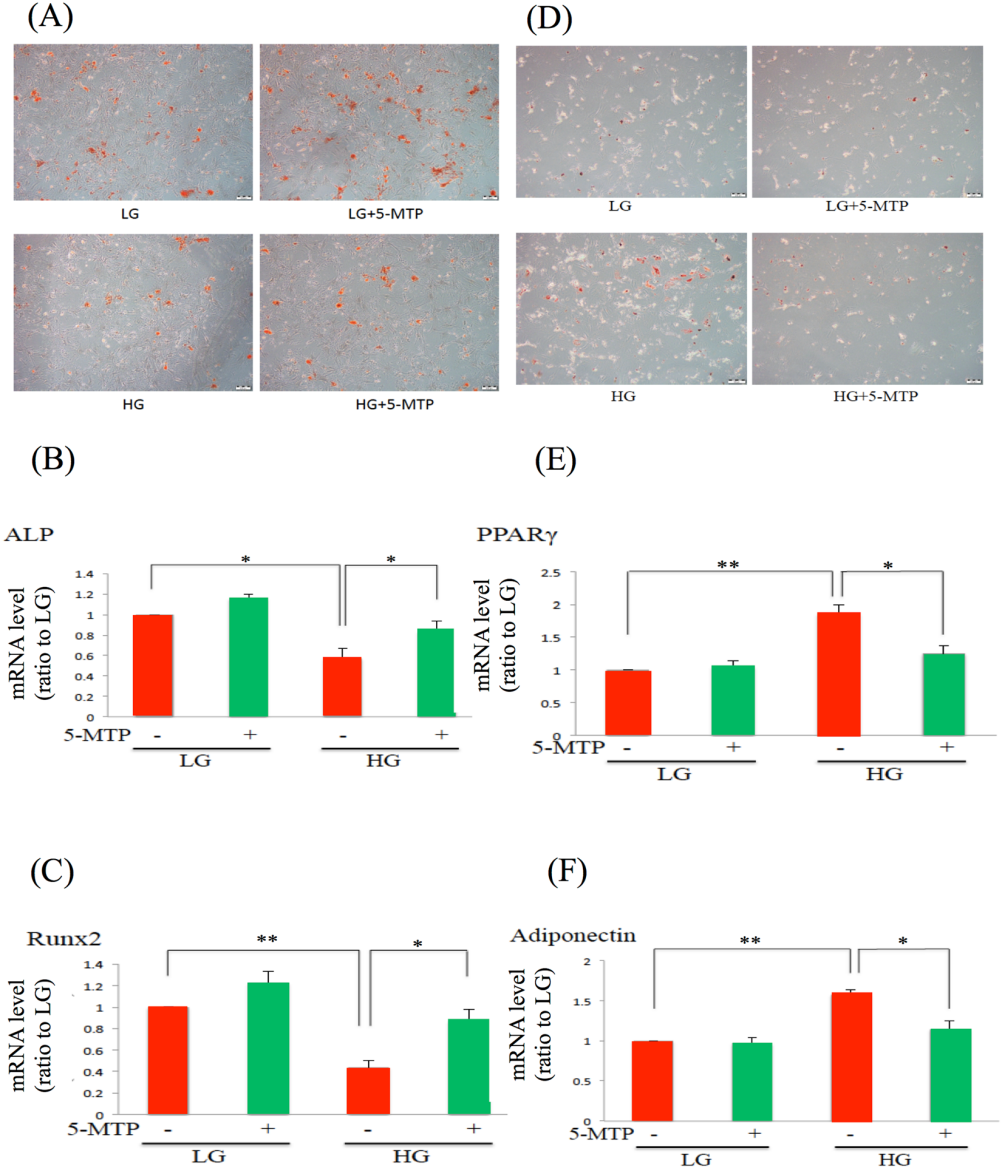
5-MTP restores osteogenic and suppresses adipogenic differentiation. BM-MSCs were incubated in LG or HG medium for 2 weeks. They were washed and incubated with osteogenesis-or adipogenesis-induction medium with or without 10 μM 5-MTP for 3 weeks. **(A**–**C)** Cells were analyzed for osteogenesis. (**A**) Alizalin red stain, (**B**) alkaline phosphatase (ALP) mRNA and (**C**) Runx2 mRNA. **(D**–**F)** Cells were analyzed for adipose differentiation. (**D**) Oil red O stain, (**E**) PPARγ mRNA and (**F**) adiponectin mRNA. mRNA expression was analyzed by qPCR. Error bars in all figures denote means ± SEM (n = 3). *p < 0.05. **p < 0.01.

As MSC differentiation potential is influenced by stress-induced cell senescence, we determined whether 5-MTP protects BM-MSC against inappropriate differentiation. Osteogenic and adipogenic differentiation is analyzed by using selective staining and molecular markers. BM-MSCs grown in HG medium exhibited reduced osteogenic differentiation as shown by reduced Alizalin Red staining (Fig. 3A), reduced expression of alkaline phosphatase (ALP) (Fig. 3B) and Runx2 (Fig. 3C), but enhanced adipogeinc differentiation as shown by increased oil red O staining (Fig. 3D) and increased expression of PPARγ (Fig. 3E) and adiponectin (Fig. 3F). 5-MTP pretreatment did not influence Alizalin Red staining of LG-MSCs but increased osteogenic differentiation of HG-MSCs (Fig. 3A). The staining data are supported by molecular markers of osteogenic differentiation. 5-MTP prevented drop of alkaline phosphatase (ALP) and RunX2 expression in HG-MSCs (Fig. 3B and C). Conversely, 5-MTP reduced oil red O adipocyte staining of HG-MSCs with corresponding control of PPARγ and adiponectin expression (Fig. 3D,E and F). These results suggest that 5-MTP coordinately regulates MSC differentiation and senescence induced by HG.

### 5-MTP attenuates H_2_O_2_-induced senescence

**Figure 4:**
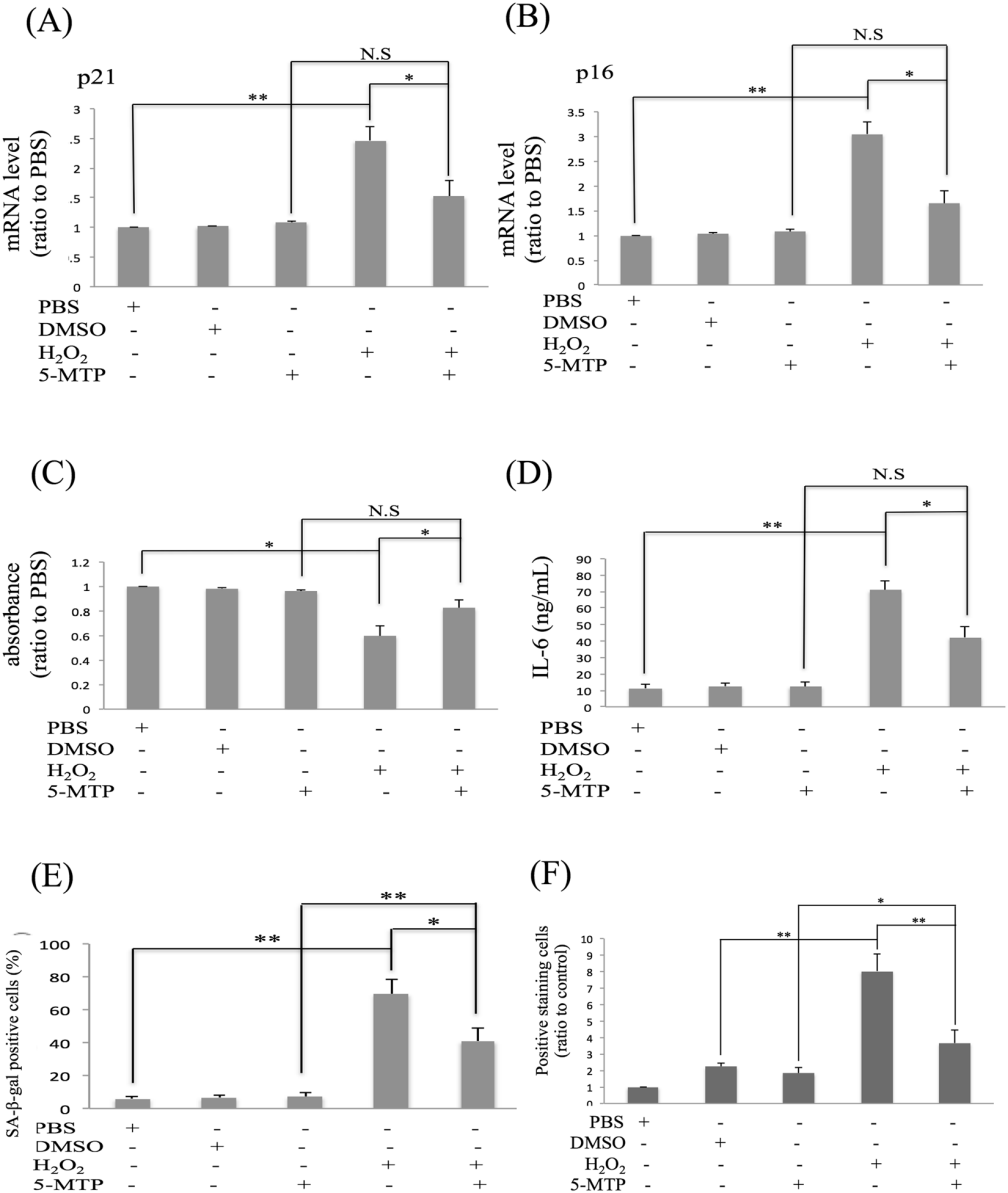
5-MTP protects BM-MSCs from H_2_O_2_-induced senescence. **(A)** p21 and **(B)** p16 mRNA levels were analyzed by qPCR. **(C)** BM-MSC growth was analyzed by BrdU incorporation. **(D)** IL-6 released in the medium was measured with ELISA. **(E)** Cells with positive SA-β-Gal staining were counted and quantitatively analyzed. **(F)** Lysotracker staining of BM-MSCs treated with H_2_O_2_ (100 μM) in the presence or absence of 5-MTP (10 μM). Lysotracker staining was quantitatively analyzed. Error bars in all figures denote means ± SEM (n = 3). *p < 0.05. **p < 0.01.

To determine whether 5-MTP exerts a direct effect on oxidant-induced premature senescence, we selected experimentally a H_2_O_2_ concentration which induces BM-MSC senescence without causing overt cell death. H_2_O_2_ at 100 μM did not alter BM-MSC survival or apoptosis as shown by MTT assay and cleaved PARP Western blotting (Supplementary Fig. S4A and B). H_2_O_2_ at the sub-lethal concentration increased p21 (Fig. 4A) and p16 (Fig. 4B) expression, reduced BrdU incorporation (Fig. 4C) and enhanced IL-6 secretion (Fig. 4D). It increased SA-β-Gal-positive (Fig. 4E and Supplementary Fig. S5) and Lysotracker-positive cells (Fig. 4F and Supplementary Fig. S6). Pretreatment of BM-MSCs with 5-MTP attenuated p16 and p21 expression (Fig. 4A and B), BrdU incorporation (Fig. 4C), IL-6 secretion (Fig. 4D), SA-β-Gal (Fig. 4E and Supplementary Fig. S5) and Lysotracker-positive cells (Fig. 4F and Supplementary Fig. S6). These results indicate that 5-MTP is effective in controlling oxidant-induced premature senescence.

### 5-MTP upregulates FOXO3a expression and increases superoxide dismutase (SOD) and Catalase activities

**Figure 5:**
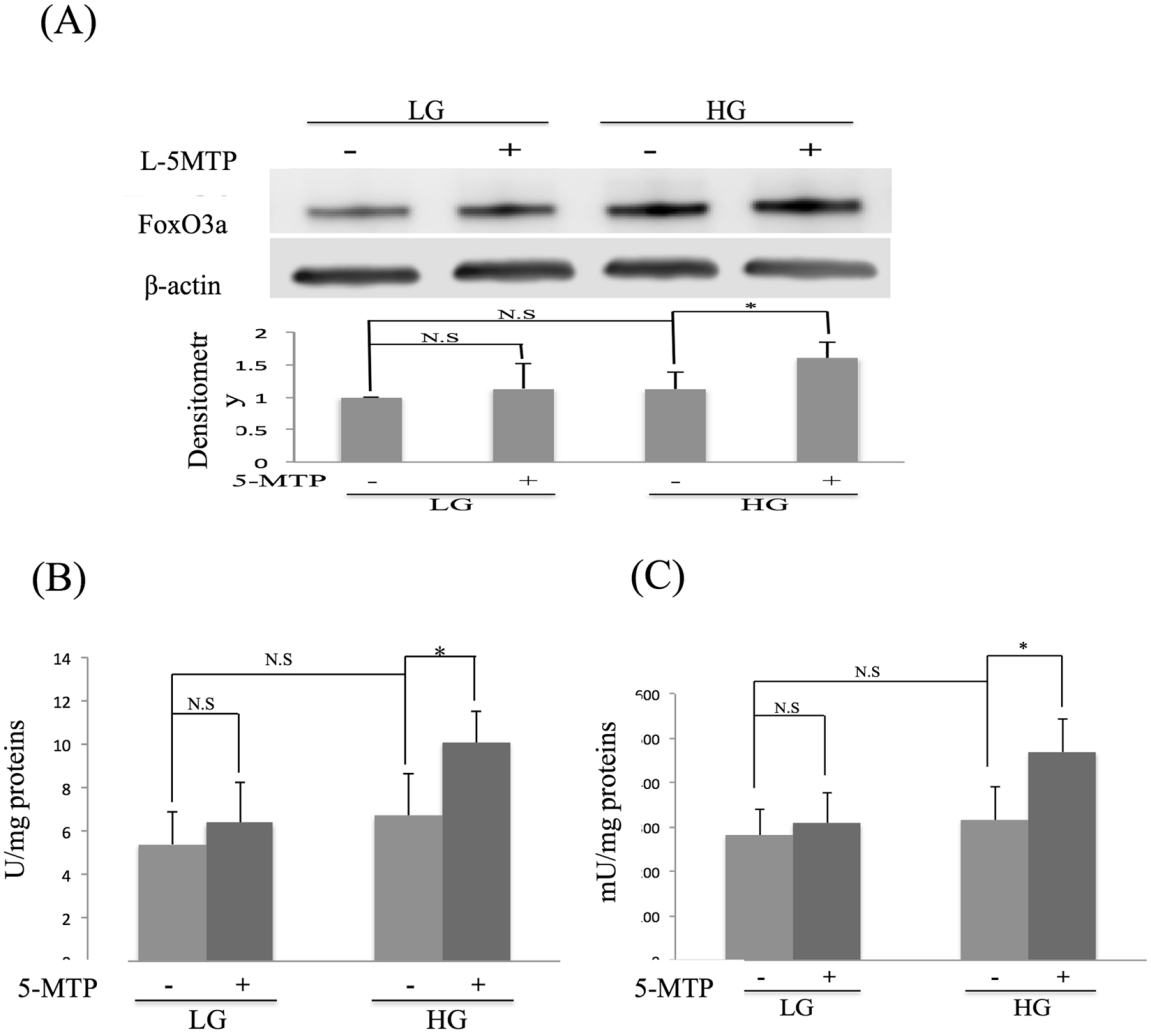
5-MTP upregulates FoxO3a and increases SOD and catalase activities. **(A)** FoxO3a proteins in LG-BM-MSCs and HG-BM-MSCs in the presence and absence of 5-MTP (10 μM) were analyzed by Western blotting. Upper panel shows representative blots and the lower panel, densitometric analysis of blots. Error bars denote mean ± SEM (n = 3). *p < 0.05. **(B)** SOD and **(C)** catalase activity in BM-MSCs incubated in LG or HG with or without 5-MTP. Error bars denote mean ± SEM (n = 3). *p < 0.05. **p < 0.01. N.S. non-significant.

**Figure 6:**
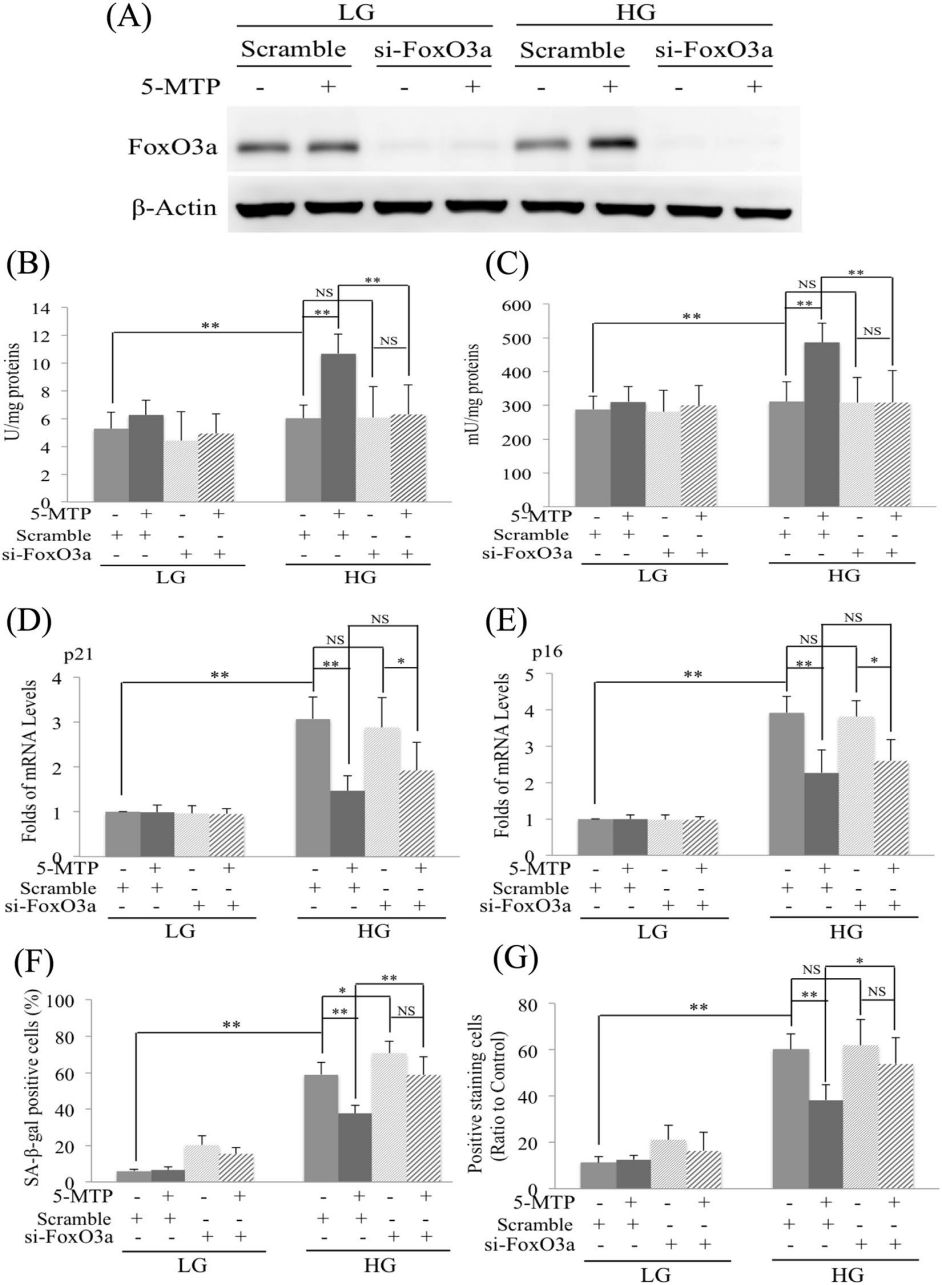
FoxO3a siRNA attenuates 5-MTP-mediated inhibition of senescence. BM-MSCs were incubated in LG-or HG- medium for 2 weeks. FoxO3a siRNA was transfected into cells for 12 h followed by 5-MTP for additional 48 h. **(A)** FoxO3a proteins were analyzed by Western blotting. **(B)** SOD and **(C)** catalase activity in BM-MSCs were measured under the indicated condition. **(D)** p16 and **(E)** p21 mRNA in BM-MSCs were analyzed with qPCR. **(F)** BM-MSCs were stained for SA-β-gal and SA-β-Gal**-**positive cells were counted under light microscopy. **(G)** IL-6 concentration in culture medium of BM-MSCs was measured with ELISA. Scramble indicated control scramble siRNA. Error bars in all figures denote means ± SEM (n = 3). *p < 0.05. **p < 0.01. N.S. non-significant.

Cellular senescence is regulated at the transcriptional level by transactivators notably NF-κB and FoxO3a. Several studies reported that NF-κB activation mediates cell senescence especially senescence-associated secretory phenotype (SASP)^9, 10^. We determined whether 5-MTP controls senescence via NF-κB inactivation. P65 was increased in cytosolic and nuclear fractions of BM-MSC grown in HG compared to those grown in LG. 5-MTP pretreatment did not reduce p65 in cytosolic or nuclear fractions of HG-MSCs (see Supplementary Fig. S7). These results suggest that the anti-senescence effect of 5-MTP is not mediated via NF-κB suppression. We next determined whether 5-MTP affects FoxO3a. HG slightly increased FoxO3a which did not reach statistical significance (Fig. 5A). 5-MTP significantly increased FoxO3a proteins in HG-MSC (Fig. 5A and Supplementary Fig. S8). As FoxO3a was reported to promote the expression of anti-oxidant genes^11^, we evaluated the effect of 5-MTP on superoxide dismutase (SOD) and catalase activities. 5-MTP significantly increased SOD (Fig. 5B) and catalase activity (Fig. 5C) in HG-MSCs. The extent of stimulation was correlated with increase in FoxO3a. These results suggest that 5-MTP enhances the anti-oxidant activities via FoxO3a upregulation in HG-MSCs. To provide direct evidence for this, we evaluated the effect of FoxO3a silencing on SOD and catalase activities. FoxO3a siRNA transfection completely blocked FoxO3a protein expression in BM-MSCs while control scRNA had no effect (Fig. 6A and Supplementary Fig. S9). FoxO3a siRNA suppressed FoxO3a proteins not only in BM-MSCs cultured in LG but also in HG (Fig. 6A and Supplementary Fig. S9). FoxO3a siRNA transfection abrogated 5-MTP-induced increase in SOD and catalase activities in HG-MSCs (Fig. 6B and C). These results indicate that 5-MTP enhances anti-oxidant actions via FoxO3a upregulation in HG-MSCs.

### FoxO3a silencing abrogates the effect of 5-MTP on SA-β-gal but not p21 or p16 in HG-stress BM-MSCs

The effect of 5-MTP on reducing HG-induced p21 and p16 elevation was not significantly influenced by FoxO3a siRNA transfection (Fig. 6D and E). By contrast, the effect of 5-MTP on lowering SA-β-Gal positive cells was reversed by FoxO3a siRNA (Fig. 6F). Interestingly, lysotracker-positive cells were similarly reversed by FoxO3a siRNA (Fig. 6G). These results suggest that the anti-oxidant enzymes induced by 5-MTP in HG-MSCs controls lysosome biogenesis and SA-β-Gal and are less effective in regulating the expression of cell cycle inhibitors.

### The anti-senescence action of 5-MTP depends on mTOR

**Figure 7:**
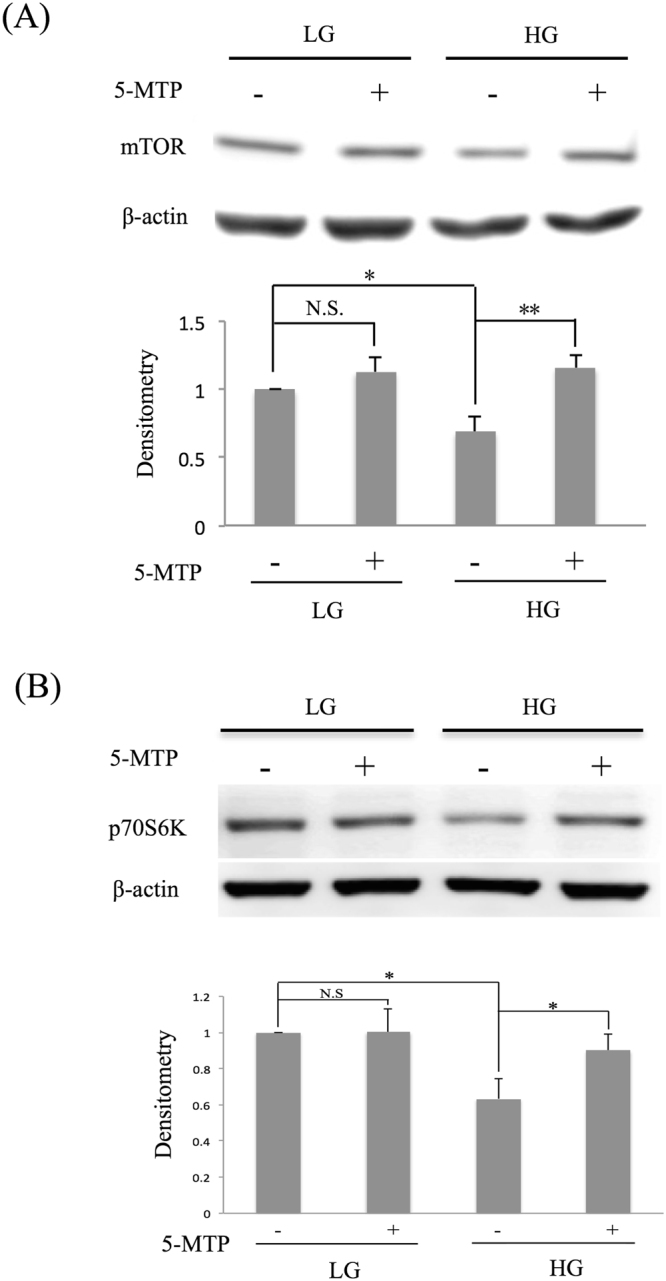
5-MTP prevents HG-induced suppression of mTOR. BM-MSCs were cultured in LG- or HG medium for 2 weeks. 5-MTP (10 μM) was added for 48 h and cells were lysed and **(A)** mTOR proteins and **(B)** p70S6 kinase were analyzed by western blotting. Upper panel shows representative blots and the lower panel the densitometric analysis. Error bars denote mean ± SEM (n = 3). *p < 0.05. **p < 0.01.

**Figure 8:**
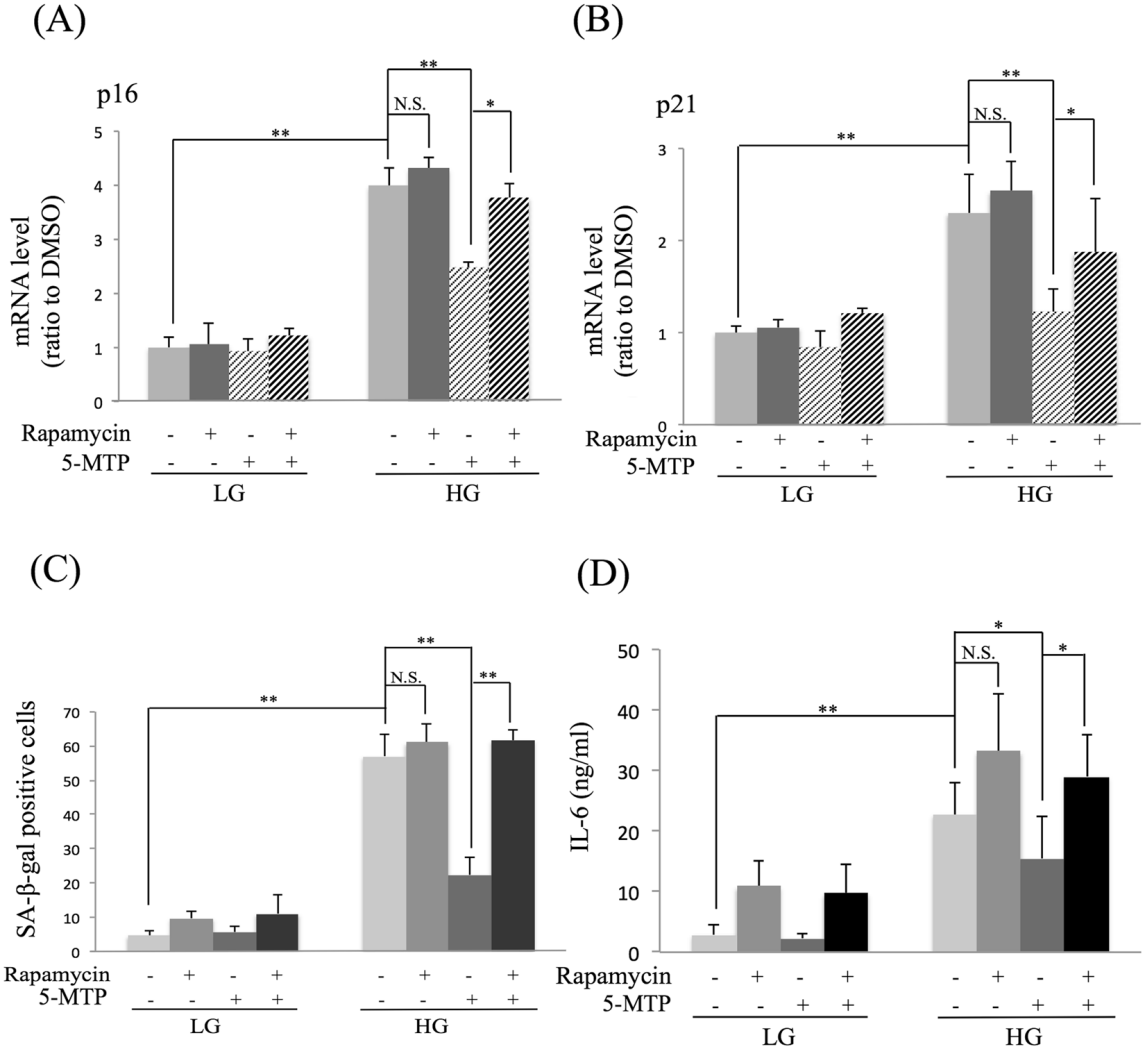
Rapamycin abrogates 5-MTP-mediated protection against senescence. BM-MSCs were incubated in LG-or HG- medium for 2 weeks. Rapamycin (5 μM) was added for 24 h followed by 5-MTP for additional 48 h. **(A)** p16 and **(B)** p21 mRNA in BM-MSCs were analyzed with qPCR. **(C)** BM-MSCs were stained for SA-β-gal and SA-β-Gal**-**positive cells were counted under light microscopy. **(D)** IL-6 concentration in culture medium of BM-MSCs was measured with ELISA. Error bars in all figures denote means ± SEM (n = 3). *p < 0.05. **p < 0.01. N.S. non-significant.

To discern the signaling pathway via which 5-MTP inhibits HG-induces BM-MSC senescence, we investigated the involvement of mechanistic target of rapamycin (mTOR). We analyzed mTOR proteins in LG- and HG-MSC. mTOR protein in HG-MSCs was significantly reduced when compared to that in LG-MSCs (Fig. 7A). 5-MTP restored mTOR proteins in HG-MSCs but did not alter mTOR proteins in LG-MSCs (Fig. 7A and Supplementary Fig. S10A). We next evaluated the effect of 5-MTP on p70 S6 Kinase (S6K). S6K activation as analyzed with phosphor-S6K was significantly reduced in HG- vs LG-BM-MSCs which was restored by 5-MTP (Fig. 7B and Supplementary Fig. S10B). To ascertain that 5-MTP inhibits senescence through mTOR, we treated BM-MSCs with rapamycin and analyzed senescence markers. Rapamycin did not have a significant effect on p16 expression in LG-MSC but abrogated the p16 lowering effect of 5-MTP in HG-MSC (Fig. 8A). Similarly, 5-MTP-induced reduction of p21 transcript was abrogated by rapamycin (Fig. 8B). Rapamycin did not influence HG-induced SA-β-Gal but abrogated the 5-MTP-mediated control of SA-β-Gal in HG-MSC (Fig. 8C). Rapamycin abrogated 5-MTP-mediated control of IL-6 in a manner similar to its abrogation of the rise of other senescence markers (Fig. 8D). These results indicate that mTOR pathway is crucial in mediating the anti-senescence effect of 5-MTP.

## Discussion

A key finding of this study is effective control of metabolic and oxidative stress-induced BM-MSC senescence by 5-MTP, a newly discovered bioactive tryptophan metabolite. 5-MTP blocks stress-induced elevation of p16 and p21 and overcomes senescent cell growth arrest. It suppresses lysosome biogenesis and SA-β-Gal and inhibits IL-6 secretion. Furthermore, 5-MTP reverses aberrant MSC differentiation caused by HG and H_2_O_2_. Thus, 5-MTP represents a new class of compounds capable of preventing oxidative stress-and HG-induced premature senescence. 5-MTP is endogenously produced in several types of human cells including fibroblasts and endothelial cells^5, 6^. It is unclear whether MSCs possess the enzymes to synthesize 5-MTP. Work is in progress in our laboratory to address this. Nevertheless, 5-MTP could potentially function as a physiological regulator of MSC senescence and has the potential to serve as a lead compound for controlling MSC senescence.

This study provides new information about the mechanism of action of 5-MTP. We show that 5-MTP upregulates FoxO3a expression in BM-MSCs treated with HG and increases the activities of anti-oxidant enzymes such as SOD and catalase. FoxO3 belongs to the Forkhead box O (Fox O) family^11, 12^. It contains DNA binding domain which interacts with the promoter region of diverse genes and regulates the expression of genes including SOD-2 and catalase^13, 14^. FoxO3a activity is regulated at transcriptional and post-translational level; phosphorylation, acetylation and ubiquitination influence FoxO3a DNA binding and transactivation of genes^15^. FoxO3a was reported to reduce cell senescence *in vitro* and confers longevity in animal models *in vivo* by increasing anti-oxidant enzymes^16–18^. Oxidative stresses were reported to disrupt the FoxO3a-dependent anti-oxidant defense mechanism and thereby induce cell cycle arrest and cell death^19, 20^. We confirm that upregulation of FoxO3a expression is accompanied by increased SOD and catalase activities. Silencing FoxO3a expression by specific siRNAs abrogates 5-MTP-induced increase in SOD and catalase activities which results in reversal of SA-β-Gal and IL-6 secretion. However, silencing FoxO3a has no significant effect on 5-MTP-induced reduction of p16 and p21. Our findings suggest that FoxO3a upregulation by 5-MTP controls a subset of HG-induced BM-MSC senescent phenotypes including SA-β-Gal and SASP through enhancement of anti-oxidant enzyme activities and reduction of reactive oxygen species.

High-glucose medium was reported to accelerate premature cellular senescence through p300 upregulation^21, 22^. P300 upregulation results in reduced SIRT and FoxO1 and the consequent reduction of SOD-2 expression^21^. Furthermore, p300 histone acetyltransferase (HAT) acetylates FoxO proteins to disrupt binding of FoxO to DNA^23^. P300 HAT was reported to down-regulate FoxO3a in muscle^24^. We have reported that 5-MTP inhibits p300 HAT and thereby reduces binding of several transactivators such as NF-κB, C/EBPβ, AP-1 and CREB to their respective motifs on COX-2 promoter resulting in down-regulation of COX-2 expression in fibroblasts^25^. It is possible that 5-MTP increases FoxO3a activity by blocking p300 HAT.

We found that HG stress suppresses mTOR protein expression which is reversed by 5-MTP. Thus, 5-MTP maintains a normal cellular level of mTOR in BM-MSCs despite stressful conditions. This 5-MTP action is crucial for preventing stress-induced senescence as inhibition of mTOR activity with rapamycin abolishes the anti-senescence effect of 5-MTP. Our findings are contrary to several reported data which indicate that mTOR is a major mediator of cellular senescence and aging^26, 27^. On the other hand, our results are consistent with a recent report which suggests that mTOR inactivation plays an essential role in autophagy-dependent senescence in murine fibrosarcoma L929 cells^28, 29^. We previously reported that BM-MSCs cultured in HG-containing medium undergo senescence in an autophagy-dependent manner^1^. It is therefore possible that mTOR may transmit opposite message in senescence depending on the content of stresses and the cell types. This notion is supported by the opposite effects of rapamycin on cell senescence and SASP. Rapamycin was reported to protect cells from senescence^30^ and block SASP, i.e. IL-6 secretion by inhibiting mTOR activity^31^. In fact, rapamycin is considered to be a candidate for enhancing longevity in mammals^32, 33^. However, our results are opposite to the reported data in that rapamycin increases BM-MSC IL-6 secretion and SA-β-Gal and abolishes the effect of 5-MTP on senescence control. We propose that in the context HG-induced BM-MSC senescence which is triggered by reduced mTOR and increased autophagy, rapamycin does not protect against senescence and SASP as it does in the other all types under different stresses.

We have previously reported that pro-inflammatory mediators such as cytokines (IL-1β and TNFα) or lipopolysaccharide (LPS) suppress endothelial 5-MTP production which contributes to uncontrolled endothelial permeability, macrophage activation and systemic inflammation^6, 7^. Intracellular 5-MTP in human umbilical vein endothelial cells was visualized by immunofluorescent microscopy. It was distributed at perinuclear endoplasmic reticulum and secreted via the ER-Golgi vesicular transport^6^. LPS reduces intracellular 5-MTP staining and 5-MTP secretion^6^. Our preliminary data suggest that 5-MTP was visualized in BM-MSCs with similar ER distribution and the intensity of staining was reduced in BM-MSCs cultured in HG (data not shown). It is possible that MSCs joins fibroblasts and endothelial cells as a source of 5-MTP production and release. 5-MTP may thus play an auto-protective role to maintain MSC homeostasis and prevent premature senescence. Under metabolic and/or oxidative stress, 5-MTP may be suppressed and the defense mechanism may be lost leading to premature senescence and secretory phenotype. Exogenous supplement of synthetic 5-MTP restores 5-MTP function. This hypothesis is currently being tested in our laboratory.

## Methods

### Materials

L-5-MTP was custom-synthesized by AstaTech (AstaTech Inc, Bristol, PA, USA). The synthesized L-5-MTP was analyzed by LC-MS and proved to be homogenous. L-5-MTP stock solution was prepared by dissolving the synthetic powder in DMSO in brown tube and stored at −20 °C or −80 °C. Rapamycin and H_2_O_2_ were obtained from Sigma-Aldrich. Human osteogenic and adipogenic differentiation reagents were purchased from R&D systems.

### Cell culture

Human bone marrow-derived MSCs (BM-MSCs) were obtained from Lonza. They were routinely cultured in Dulbecco’s modified Eagle’s medium (DMEM) containing 1.0 g/L glucose (5.5 mM) and HEPES (25 mM) and supplemented with 10% fetal bovine serum (Hyclone), 100 U/ml penicillin and 100 μg/ml streptomycin at 37 °C in a humidified 5% CO_2_ atmosphere. For this study, cultured BM-MSCs were washed and divided into two groups: one group was cultured in regular medium and the other in high-glucose (HG) (4.5 g/L, 25 μM). In all experiments of H_2_O_2_-induced senescence, cells were treated with 2 pauses of H_2_O_2_ (100 μM) for 4 h, washed and incubated in fresh medium without H_2_O_2_ for 92 h after each pause. To evaluate the effect of 5-MTP on H_2_O_2_-induced senescence, 5-MTP (10 μM) was added and incubated for 48 h before cell harvest after the second pause.

### BrdU incorporation analysis

Cell proliferation was analyzed with bromodeoxyuridine (BrdU) assay kit (Chemicon). In brief, cells were incubated with 20 μM of BrdU before cells were harvested. They were then treated with HCS fixation solution (1X) at room temperature (RT) for 30 min followed by mouse anti-BrdU antibody for 1 hr, After washing, goat anti-mouse IgG was added and kept at RT for 1 hr. Cells were washed and incubated with TMB peroxidase substrate for 30 min. The reaction was stopped and the sample was analyzed with a multimode microplate reader at 450 nm.

### Quantitative real-time PCR (qPCR) analysis

Total RNA was isolated using TRIzol reagent (Invitrogen, Paisely, Scotland) according to the manufacturer’s instructions. RNA concentration was quantified with a NanoDrop ND-1000 Spectrophotometer (Nanodrop Technologies) and RNA quality was checked with the ratio of OD 260/280. Quantification of mRNA expression for candidate genes was performed by qPCR using ABI One-step Detection System Instrument (Applied Biosystems). Total RNA was reverse-transcribed by using high capacity cDNA reverse transcription kit (Invitrogen). qPCR reactions were performed with the power SYBR Green PCR Master mix (Roche) in a MicroAmp optical 96-well reaction plate according to the manufacturer’s instructions. Relative gene expression levels were normalized to GAPDH expression. The primer sequences of each gene for qPCR were as follows. p21: forward primer (F), 5′- AGT G G A A T T A G C C C T C A G C A-3′and reverse primer (R), 5′-C A T G G T C C C TG G G T T C T T C-3′; p16: F, 5′-C T A C T G A G G A G C C A G C G T C T-3′ and R, 5′-C T G CCCATCATCATGACCT-3′and GADPH: F, 5′-GAAATCCCATCACCATCTTCCAGG-3′; and R, 5′-GAGCCCCAGCCTTCTCCATG-3′. Positive control for p21 was performed in A549 cells treated with etoposide^34^. HeLa cells and SKOV3 cells were selected as p16 positive and negative controls^35, 36^.

### Measurement of IL-6

IL-6 in the medium of cultured BM-MSCs was determined with an ELISA kit (Abcam) according to the manufacturer’s instructions. Briefly, a monoclonal antibody specific for IL-6 was coated onto the wells of microtiter plates. Samples and IL-6 standards were pipetted into wells. After incubation and washing, biotinylated monoclonal antibody specific for IL-6 was added, followed by streptavidin-HRP and TMB substrate. A standard curve was constructed and IL-6 concentrations in the samples were measured.

### Senescence associated β-galactosidase staining

Expression of pH-dependent senescence associated β-galactosidase (SA-β-gal) in BM-MSCs was analyzed using a SA-β-gal staining kit (Cell Signaling Technology) according to manufacturer’s instruction. Briefly, BM-MSCs grown in a microwell plate were washed and incubated in 1X fixative solution. After washing two times with PBS, β-Gal staining solution (final concentration 1 mg/mL, pH 6.0) was added to each well. The plate was sealed with parafilm to prevent evaporation and incubated at 37 °C overnight. Cells were examined under a light microscope. We counted total cell numbers and number of cells with positive blue staining in each well. The results were expressed as percentage of positive stained cells. BM-MSCs undergoing replicative senescence and A549 cells were included as positive and negative controls, respectively (Figure S2).

### Lysotracker stain

Lysosomes were labeled by LysoTracker Red DND 99 (Molecular Probes, by Life Technologies) according to manufacture instruction. In brief, BM-MSCs were washed and incubated in prewarmed (37 °C) probe-containing medium for 2 h at 37 °C in 5% CO_2_. Probe-containing medium was prepared by diluting 1 mM probe stock solution to the final working concentration of 50 nM in the growth medium. Following incubation, cells were washed, incubated in PBS and examined under fluorescent microscopy fitted with a suitable filter set. Cells containing red fluorescent dots were detected and analyzed with Image J software. BM-MSCs grown in LG medium pretreated with bafilomycin were included as negative control for Lysotracker staining and cells treated with rapamycin were included as positive control (Figure S3).

### Osteogenic and adipogenic differentiation

Osteogenic and adipogenic differentiation was performed using StemXVivo osteogenic and adipogenic differentiation media (R&D systems). 5 × 10^4^ cells were seeded to each well of a 6-well plate with StemXVivo Osteogenic/Adipogenic Base Medium. When cells grew to 70% confluency, base medium were replaced by differentiation medium with StemXVivo Osteogenic/Adipogenic Supplements. Differentiation medium were replaced with fresh differentiation medium once every 3–4 days. After 21 days, osteoblasts were fixed with 70% ethanol and stained with 2% Alizalin Red; adipocytes were fixed with 10% formalin and stained with oil-red O, respectively. Cells were washed with ddH_2_O and examined under light microscopy.

### Western blot analysis

Western blotting was performed as previously described [1]. Rabbit polyclonal antibody against mTOR, p65 and PARP and rabbit FoxO3a and p70S6K kinase mAb were purchased from Cell Signaling. Mouse monoclonal antibodies against β-actin were obtained from Sigma.

### SOD and catalase activity assay

SOD activity assay was performed with SOD activity assay kit from Abcam. 2 × 10^6^ cells are lysed and centrifuged at 14,000x g for 5 minutes at 4 °C. Supernatants were collected. 20 μL of supernatant was mixed with enzyme working solution (with superoxide), WST working solution (with WST to detect superoxide dot) and ddH_2_O incubated at 37 °C for 20 minutes. The optical density of the samples was measured at OD 450 nm in a microplate reader.

Catalase activity was assayed with catalase assay kit from Abcam. 2 × 10^6^ cells are lysed and centrifuged at 14,000 x g for 5 minutes at 4 °C. Supernatants were collected. 78 μL of the supernatant was mixed with 12 μL of catalase reaction solution (with H_2_O_2_), 2 μL of HRP solution, 2 μL of oxired probe and 46 μL assay buffer, and incubated at 25 °C for 10 minutes. The sample optical density was measured at OD 570 nm in a microplate reader.

### FoxO3a siRNA transfection

Two FoxO3a siRNA (ID no. s5261 and s5262) were designed and synthesized by Ambion (Invitrogen). Sequences of FoxO3a siRNA are 5′-UGACAGAAUUCGACAAGGCAC-3′ and 5′-UUGAGUACAAGGAGGAGGAGCCTG-3′. These two siRNA were mixed and transfected into BM-MSCs with Lipofectamine RNAiMAX (Invitrogen) according to manufacture instruction. In brief, BM-MSCs (1 × 10^6^ cells) were incubated with 300 μL Opti-MEM medium containing 30 pmol of FoxO3a siRNA mixture and 9 μL of RNAiMAX reagent for 48 h.

### Statistical analysis

Differences between groups were analyzed by Student *t* test. A *p* value of less than 0.05 was considered statistically significant. At least three independent experiments (n = 3) from a different set of cells and RNA isolation were performed.

## Electronic supplementary material

Supplementary Information
