# Supplementary material for: 5-methoxytryptophan protects MSCs from stress induced premature senescence by upregulating FoxO3a and mTOR
Source: Sci Rep. 2017 Sep 11;7:11133. doi: 10.1038/s41598-017-11077-4 (PMC5593915; doi:10.1038/s41598-017-11077-4)
Supplement: Supplementary file 1 — Supplementary Information [file 41598_2017_11077_MOESM1_ESM.doc]

**Supplementary Information**

**5-methoxytryptophan protects MSCs from stress induced premature senescence by upregulating FoxO3a and mTOR**

Tzu-Ching Chang^1,2^, Min-Fen Hsu^1^, Chiu-Yueh Shih^1^, Kenneth K Wu^1,2,3^

^1^Metabolomic Medicine Research Center China Medical University Hospital, Taichung, Taiwan, ^2^Graduate Institute of Biomedical Sciences, China Medical University, Taichung, Taiwan, ^3^Institute of Cellular and System Medicine, National Health Research Institutes, Zhunan Taiwan.

**Figure S1**

**(A)**

**
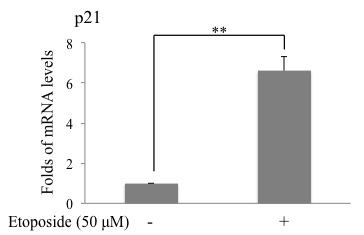
**

**(B)**

**
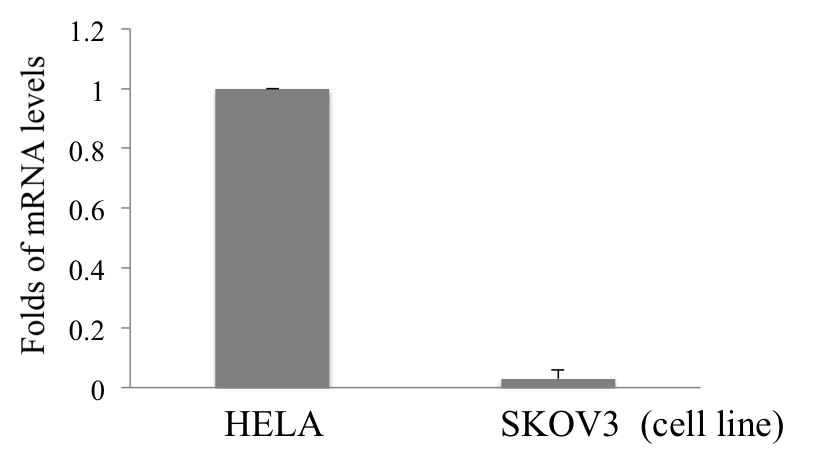
**

*Figure S1.* Positive and negative control of p21 and p16 mRNA analysis by qPCR. **(A)** A549 cells were treated with or without etoposide (50 μM) for 24 h and p21 mRNA levels in A549 cells were measured by quantitative real-time PCR. Etoposide induced p21 expression in A549 cells significantly. The error bars refer to mean ± SEM (n = 3). **(B)** HeLa cervical carcinoma cells expressing wild-type p16 were included as positive control and SKOV3 ovarian cancer cell lines with homozygous deletion of p16 were included as negative control of p16 mRNA analysis.

**Figure S2**


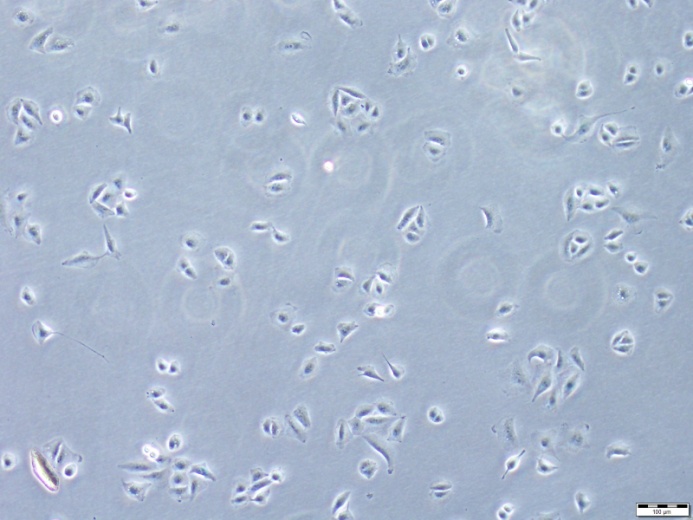

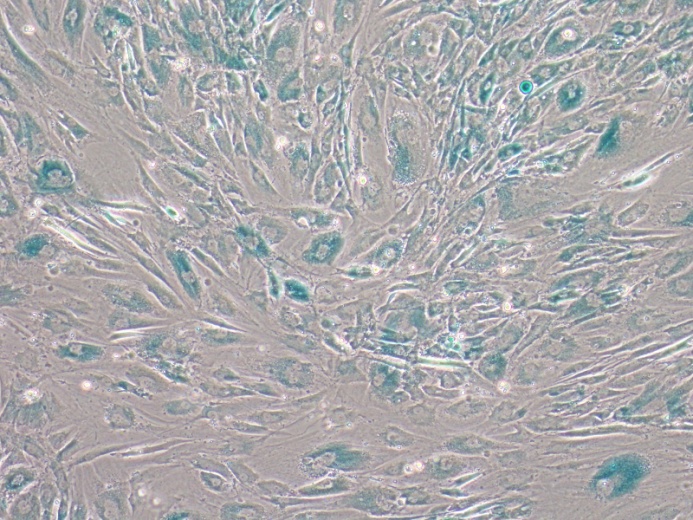


*Figure S2.* Negative and positive control of senescence associated β-galactosidase staining. Left figure showed negative control by staining non-senescent A549 cancer cell line. Right figure showed positive control by staining MSCs with replicative senescence. Replicative senescence was defected while MSCs were cultured for over 17 passages.

**Figure S3**


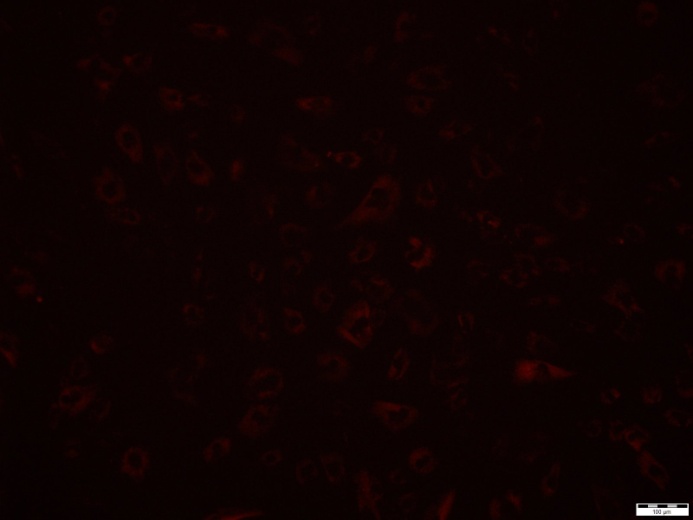

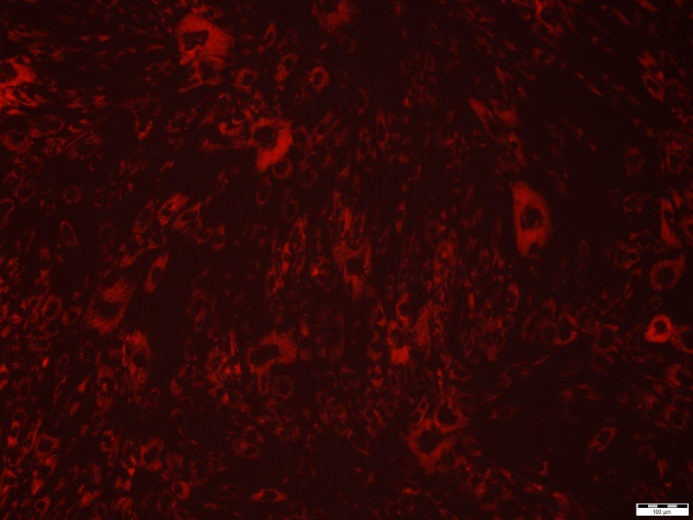


*Figure S3.* Negative and positive control of lysotracker staining. In left panel, MSCs treated with bafinomycin (5 μM), a lysosme inhibitor for 24 h, served as a negative control and in right panel, MSCs treated with rapamycin (10 μM), an autophagy and lysosome activator for 24 h, served as a positive control of lysotracker staining.

**Figure S4**

**(A)**

**
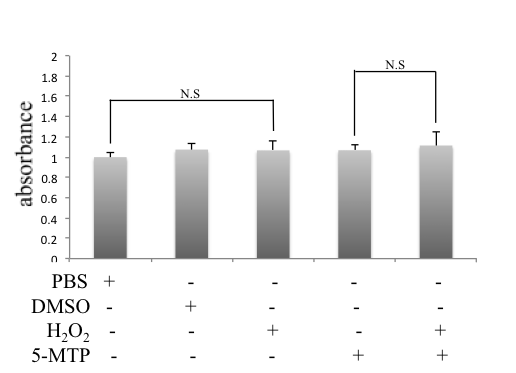
**

**(B)**

**
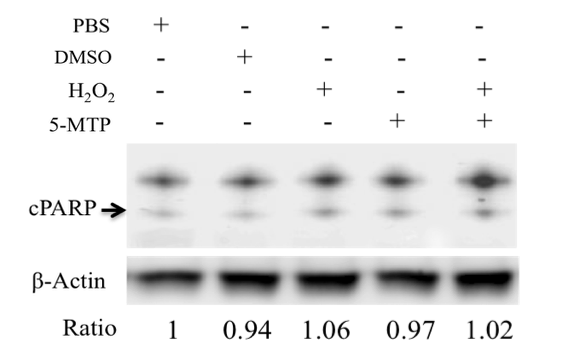
**

*Figure S4.* H_2_O_2_ at 100 μM does not induce BMMSC apoptosis**.** BM-MSC senescence was treated with two doses of H_2_O_2_ at 100 μM for 2 weeks followed by chemosynthetic pure L-5-MTP (10 μM) for 48 hrs. BM-MSCs were harvested. **(A)** Analysis of cell survival by MTT assay. **(B)** Analysis of cell apoptosis by detecting cleaved-PARP (cPARP) proteins with western blotting.

**Figure S5**

**
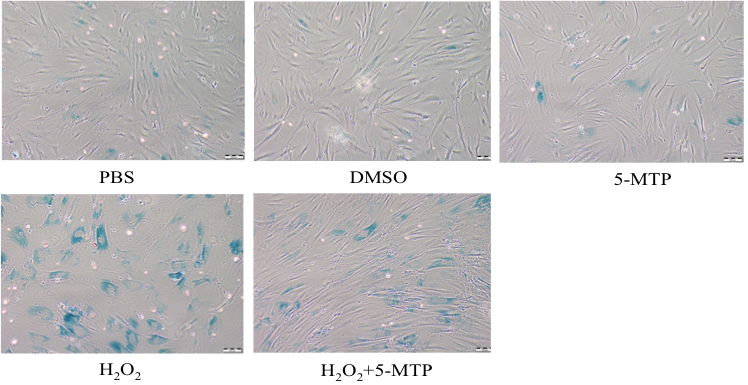
**

*Figure S5.* **5-MTP suppresses H_2_O_2_-induced SA-β-gal in BM-MSCs.** BM-MSCs treated with or without H_2_O_2_ were incubated with 5-MTP for 48 hrs. Cells were stained for SA-β-gal and examined under light microscopy. Representative figures are shown.

**Figure S6**

**
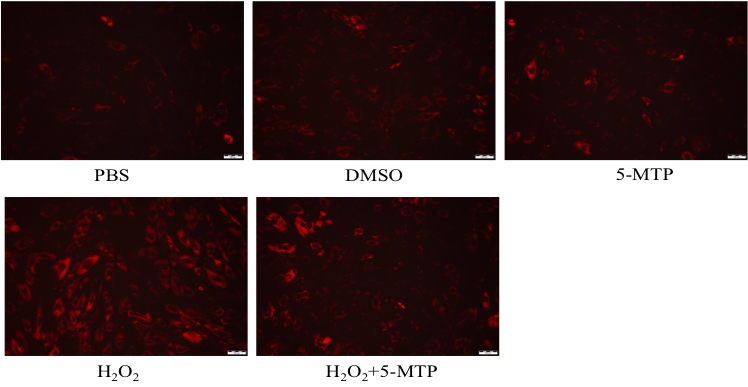
**

*Figure S6.* **5-MTP suppresses H_2_O_2_-induced lysosome expansion in BM-MSCs.** Lysotracker staining of BM-MSCs treated with H_2_O_2_ (100 μM) in the presence or absence of 5-MTP (10 μM). Representative figures are shown.

**Figure S7**


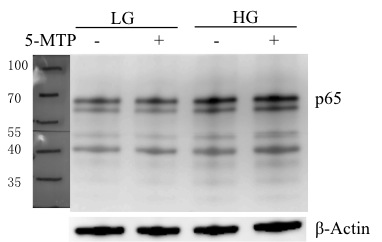

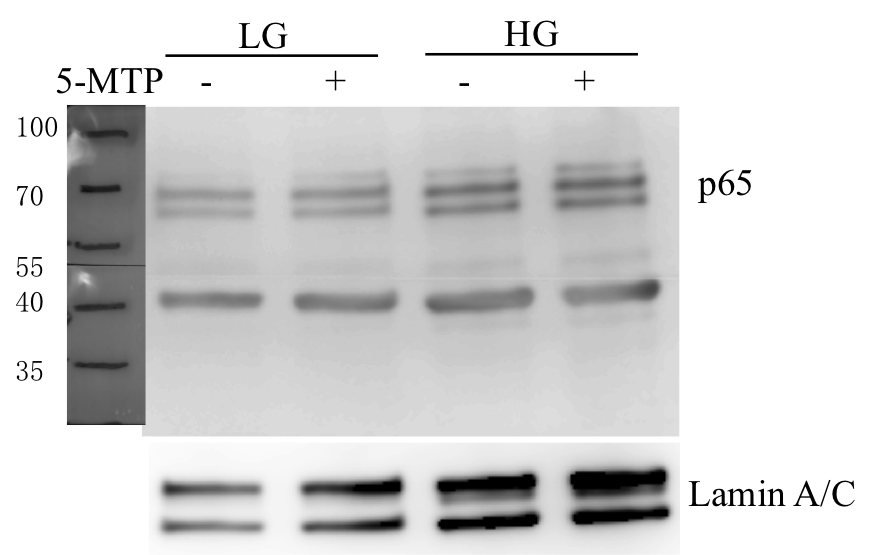


*Figure S7.* **5-MTP does not affect p65 expression and translocation in HG-induced BM-MSC senescence.** Proteins extracted from cytosolic fraction (left panel) and nuclear fraction (right panel) were analyzed by Western blotting using specific p65 antibody. β-actin and lamin A/C were included as internal loading control for cytosolic and nuclear extracts, respectively.

**Figure S8**

**
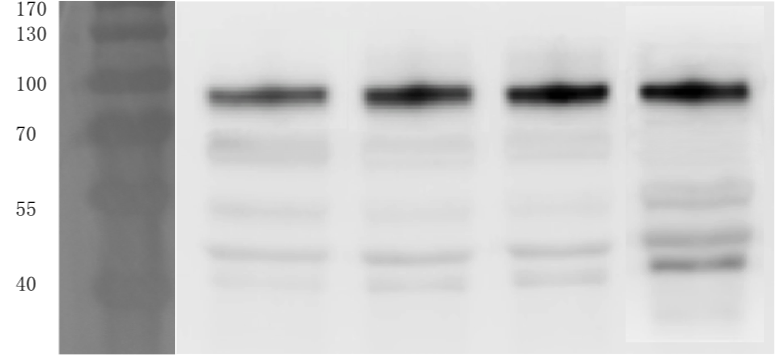
**

FoxO3a

*Figure S8.* Whole gel image of western blot of FoxO3a shown in Figure 5A.

Figure S9

**
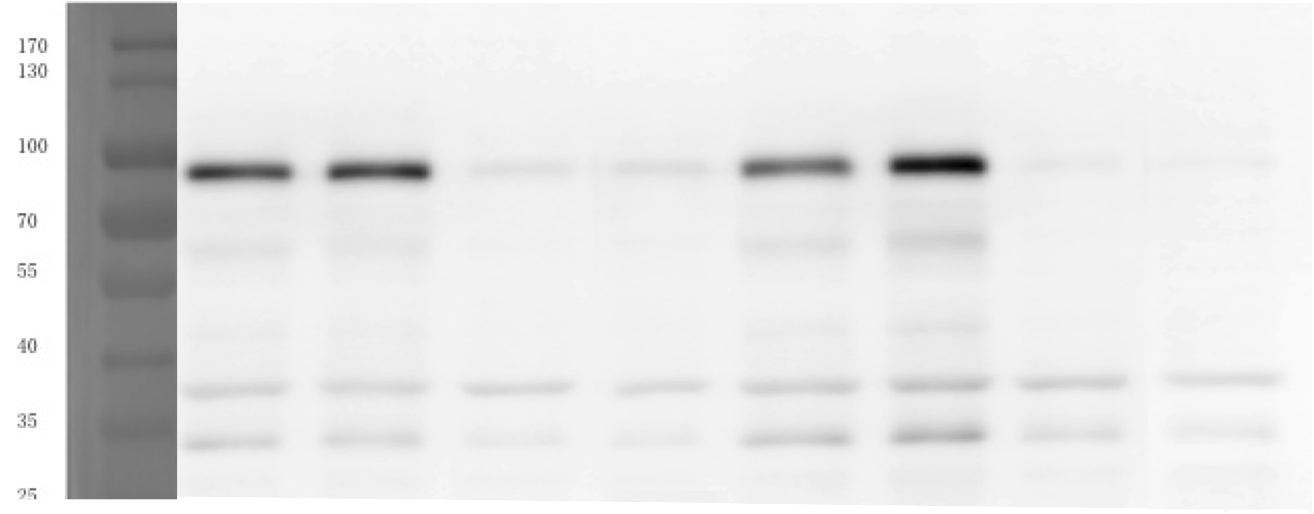
**

FoxO3a

*Figure S9.* Whole gel image of FoxO3a western shown in Figure 6A.

**Figure S10**

mTOR

**
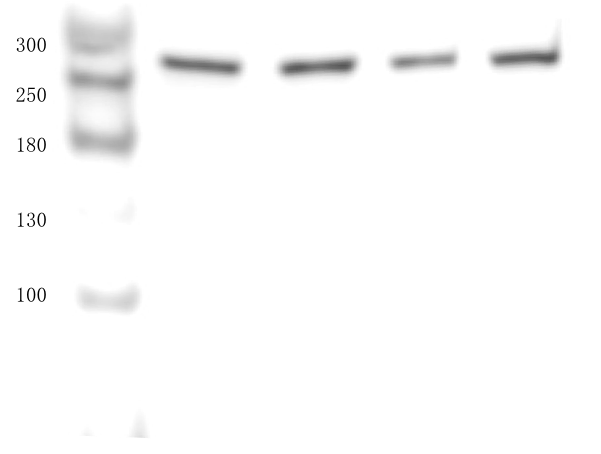
**

**
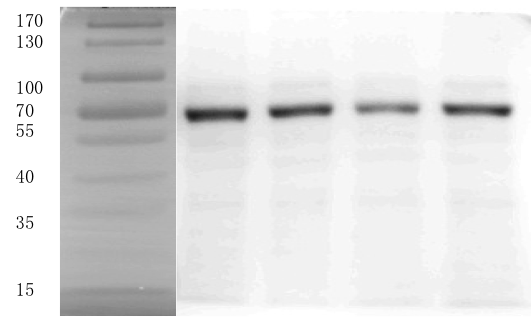
**

p70S6K

*Figure S10.* Whole gel image of mTOR and p70S6K western blotting shown in Figure 7.
